# Estimating Vitamin C Status in Critically Ill Patients with a Novel Point-of-Care Oxidation-Reduction Potential Measurement

**DOI:** 10.3390/nu11051031

**Published:** 2019-05-08

**Authors:** Sander Rozemeijer, Angélique M. E. Spoelstra-de Man, Sophie Coenen, Bob Smit, Paul W. G. Elbers, Harm-Jan de Grooth, Armand R. J. Girbes, Heleen M. Oudemans-van Straaten

**Affiliations:** 1 Department of Intensive Care Medicine, Amsterdam UMC, Location VUmc, Vrije Universiteit Amsterdam, De Boelelaan 1117, 1081 HV Amsterdam, The Netherlands; am.spoelstra@vumc.nl (A.M.E.S.-d.M.); s.coenen@vumc.nl (S.C.); b.smit@labwest.nl (B.S.); p.elbers@vumc.nl (P.W.G.E.); h.degrooth@vumc.nl (H.-J.d.G.); arj.girbes@vumc.nl (A.R.J.G.); h.oudemans@vumc.nl (H.M.O.-v.S.); 2 Research VUmc Intensive Care (REVIVE), 1081 HV Amsterdam, The Netherlands; 3 Amsterdam Cardiovascular Science (ACS), 1081 HV Amsterdam, The Netherlands; 4 Amsterdam Medical Data Science (AMDS), 1081 HV Amsterdam, The Netherlands; 5 Amsterdam Infection and Immunity Institute (AI&II), 1081 HV Amsterdam, The Netherlands

**Keywords:** vitamin C deficiency, reactive oxygen species, oxidative stress, point-of-care device, oxidation-reduction potential, antioxidant capacity, ascorbate, ascorbic acid

## Abstract

Vitamin C deficiency is common in critically ill patients. Vitamin C, the most important antioxidant, is likely consumed during oxidative stress and deficiency is associated with organ dysfunction and mortality. Assessment of vitamin C status may be important to identify patients who might benefit from vitamin C administration. Up to now, vitamin C concentrations are not available in daily clinical practice. Recently, a point-of-care device has been developed that measures the static oxidation-reduction potential (sORP), reflecting oxidative stress, and antioxidant capacity (AOC). The aim of this study was to determine whether plasma vitamin C concentrations were associated with plasma sORP and AOC. Plasma vitamin C concentration, sORP and AOC were measured in three groups: healthy volunteers, critically ill patients, and critically ill patients receiving 2- or 10-g vitamin C infusion. Its association was analyzed using regression models and by assessment of concordance. We measured 211 samples obtained from 103 subjects. Vitamin C concentrations were negatively associated with sORP (*R*^2^ = 0.816) and positively associated with AOC (*R*^2^ = 0.842). A high concordance of 94–100% was found between vitamin C concentration and sORP/AOC. Thus, plasma vitamin C concentrations are strongly associated with plasma sORP and AOC, as measured with a novel point-of-care device. Therefore, measuring sORP and AOC at the bedside has the potential to identify and monitor patients with oxidative stress and vitamin C deficiency.

## 1. Introduction

Vitamin C deficiency is common in critically ill patients with severe sepsis, trauma or ischemia/reperfusion injury and is associated with endothelial damage, cellular injury, organ dysfunction, and mortality [1,2,3]. Vitamin C (ascorbic acid) is the most important circulating antioxidant [4,5] and has pleiotropic effects, all based on the donation of its electrons [6,7,8,9]. Deficiency is likely due to increased metabolic consumption of vitamin C during oxidative stress and reduced recycling of its oxidized form dehydroascorbic acid [10,11,12]. Assessment of vitamin C status may be of interest to identify patients who might benefit from vitamin C administration and to guide its dosing [12,13]. However, vitamin C deficiency often goes unnoticed because plasma vitamin C concentrations are not available in daily clinical practice, neither is a marker of oxidative stress. The current gold-standard for vitamin C measurement involves high-pressure liquid chromatography (HPLC) with electrochemical detection, which is time consuming and not routinely performed in most hospitals. Therefore, a simple method identifying patients with low vitamin C status and oxidative stress is needed.

Recently, a point-of-care device (RedoxSYS Diagnostic System, Aytu Bioscience, Englewood, CO, USA) has been developed that allows quick determination of the static oxidation-reduction potential (sORP) and antioxidant capacity (AOC) in a clinical setting [14]. ORP (also called the redox potential) is the net balance of the activity between oxidants and reductants and reflects the amount of oxidative stress. In plasma samples that were oxidized with H_2_O_2_ (0.1%), a higher baseline sORP was found compared to non-oxidized samples. After adding ascorbic acid to the oxidized samples sORP declined to a larger extend compared to non-oxidized plasma samples [15]. High and low ORP standard solutions showed the same pattern after the addition of vitamin C [16]. Moreover, sORP was negatively related to increasing ascorbate concentrations in control solutions of buffer as well [14,17]. Furthermore, ORP was related to the degree of oxidative stress and outcome in cardiac surgical patients [18] and patients with heart failure, sepsis, and trauma [14,19,20,21,22,23,24,25].

The primary aim of this study in critically ill patients and healthy volunteers was to determine whether plasma vitamin C concentrations are associated with plasma sORP and AOC, as measured with the RedoxSYS system. The secondary aim was to determine the response of sORP and AOC to changes in vitamin C concentrations of patients receiving different doses of intravenous vitamin C.

## 2. Materials and Methods

### 2.1. Setting

The study was performed at the department of Adult Intensive Care of the Amsterdam University Medical Centers, Location VUmc, Amsterdam, the Netherlands. We performed sORP and AOC measurements in stored plasma samples collected during two studies: an observational vitamin C status study and a randomized pharmacokinetics study [2,26]. Samples were collected in 2013 and 2015, respectively, and stored at −80 °C. Plasma vitamin C concentrations have already been published. Both studies were approved by the Ethical Board of the VUmc. For the first study, the Ethical Board waived the requirement to obtain informed consent. For the second study, written informed consent was obtained from the participant or his legal representative before inclusion (registration NL50578.029.15, decision 2014.539). As part of the informed consent procedure, permission was obtained for the storage and use of residual bodily material for future scientific research.

### 2.2. Study Cohorts

#### 2.2.1. Vitamin C Status Study

Plasma vitamin C concentrations were determined in two groups of ventilator- or vasopressor dependent adult critically ill patients: patients with a systemic inflammatory response due to sepsis, major surgery or trauma and patients after cardiac arrest [2]. Heparinized blood samples were obtained for vitamin C determination on the day of admission (D1) and on day 3 (D3) if the patient remained in the intensive care unit (ICU). In addition, plasma vitamin C concentrations were measured in a group of healthy non-fasting non-smoking volunteers who did not have any dietary restrictions and did not take any vitamin supplements one month prior to the sample collection.

#### 2.2.2. Pharmacokinetic Study

This study included adult critically ill patients admitted with multiple organ failure and severe sepsis, major surgery or trauma. Patients were randomized to either 2 or 10 g of vitamin C. These dose regimens were administered either as a twice-daily bolus or as a continuous infusion for 48-h (four dose regimens in total) [26]. Inclusion was at the day of ICU-admission or later depending on the time we obtained informed consent. Heparinized blood samples were taken for vitamin C determination at enrolment (baseline) and at predefined intervals up to 96-h after the start of vitamin C supplementation. We used the following sampling time points (hours) for the present study: T0 (baseline), 1, 24, 48, and 72. Vitamin C concentrations at T24 and T48 are trough concentrations. T72 represents a wash-out phase sample. For the present analysis, we divided the patients in two groups: one group receiving the 2-g regimen and one group receiving the 10-g regimen.

### 2.3. Vitamin C Measurements

#### 2.3.1. Vitamin C Status Study

Obtained blood samples were directly processed by centrifugation (10 min, 1800 RPM). One tube with plasma was immediately stabilized with 5.0% meta-phosphoric acid (1:5) and frozen at −80 °C until vitamin C measurement in order to prevent oxidation of vitamin C in the samples. A second tube with non-acidified plasma was frozen at −80 °C as well. Plasma vitamin C concentrations were measured at the Amsterdam University Medical Centers, Location VUmc. After thawing the acidified plasma, ascorbic acid (AA) was enzymatically oxidized to dehydro-ascorbic acid (DHA) and derivatized with o-phenylenediamine. The resulting compound was separated and quantified using high-performance liquid chromatography (HPLC) with fluorescent detection. Measured plasma concentrations represent the sum of AA and DHA. Measurements showed good repeatability and reproducibility with an intra-assay coefficient of variation of 1.4% and an inter-assay coefficient of variation of 2%.

#### 2.3.2. Pharmacokinetic Study

Obtained blood samples were directly processed by centrifugation (10 min, 1800 RPM). Two tubes (one duplicate) with plasma were immediately stabilized with 5.6% meta-phosphoric acid (1:5) and frozen at −80 °C until vitamin C measurement. Plasma vitamin C concentrations were measured in the Clinical Chemistry Laboratory of the Reinier de Graaf Hospital in Delft. After thawing, vitamin C concentration was measured in one of the acidified tubes, using HPLC and electrochemical detection. In order to determine the total AA concentration by electrochemical detection, a pre-analysis reduction of in vivo formed DHA to AA was carried out. The coefficient of variation was <7%.

### 2.4. Defining Vitamin C Deficiency

Normal plasma vitamin C concentrations range from 23 to 100 µmol/L. Hypovitaminosis C and vitamin C deficiency are defined as a plasma vitamin C concentration of <23 µmol/L and <11 µmol/L, respectively [10].

### 2.5. sORP and AOC Measurements

**Figure 1:**
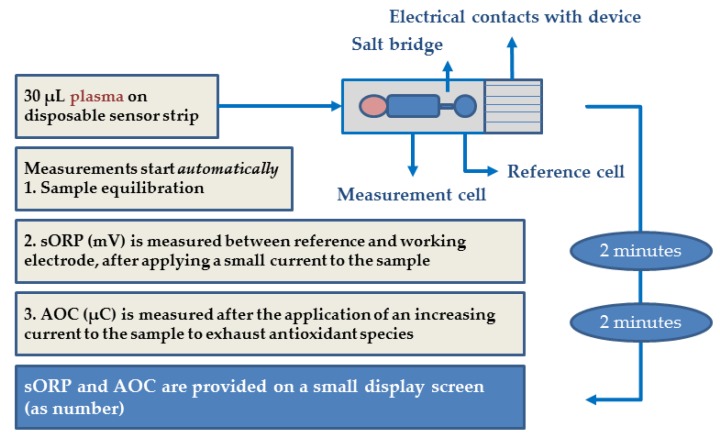
Graphic representation of the methodological process.

From both studies, the second stored plasma tubes were used to measure sORP and AOC using the RedoxSYS Diagnostic System (Aytu Bioscience, Englewood, CO, USA). The RedoxSYS Diagnostic System consists of a RedoxSYS analyzer, which provides the sORP and AOC results on a small display screen, and a single-use disposable RedoxSYS sensor strip, which consists of a working, counter and reference electrode (Figure 1) [16]. Prior to the analysis, we performed a calibration verification test of the analyzer and verified each new shipment of RedoxSYS sensors with a Zobell’s solution (Oxidation-Reduction Potential Standard). Plasma samples, which had not been thawed before, were thawed in a dark cabinet for 30–60 min at room temperature. 30 µL of the biological sample was introduced to a sensor strip, pre-inserted into a galvanostat-based reader. The static ORP is measured in less than 2 min by applying a small current to the sample and measuring the difference in potential between the working and reference electrode in mV. During the following 2 min, the current is gradually increased, which exhausts antioxidant species in the sample. The antioxidant capacity (AOC, µC) is determined by the total amount of charge required to change the ORP at maximum velocity, which indicates that all oxidizable molecules have been oxidized. In other words, the more antioxidants present in the sample, the more charge is required to oxidize them completely, which results in a higher AOC. More detailed information about the RedoxSYS system has previously been described [14,16]. Stability tests showed consistent sORP results after 6 months storage frozen at −80 °C [16]. Previous studies reported sORP values below 150 mV in heparinized plasma obtained from healthy controls [14,15,17]. One study suggests sORP values above 150 mV as an indication of oxidative stress [14]. The measurement of AOC is limited to a maximum level of capacitance of 9.13 µC, thus very high levels of AOC cannot be measured. AOC could therefore not be analyzed in plasma samples with very high vitamin C concentrations (>500 µmol/L) in the pharmacokinetic study.

### 2.6. Sample Collecting and Processing

Table 1 presents an overview of the handling of the two sample collections.

### 2.7. Statistics

We analyzed data using IBM SPSS Statistics version 22. Since acidification influences sORP, the results of both studies are presented separately because we used non-acidified samples in the vitamin C status study and acidified plasma samples in the pharmacokinetic study (see above).

Sequential vitamin C concentrations, sORP, and AOC values were compared using Paired Samples *T*-testing for normally distributed values or Wilcoxon signed rank testing for not normally distributed values. Normality was tested using assessment of skewness, histograms and the Shapiro-Wilk Test. Normally distributed variables are reported as mean ± standard deviation (SD), not normally distributed variables as median (25th to 75th) percentile. A *p*-value of <0.05 was considered to be statistically significant. Sequential vitamin C concentrations, sORP, and AOC were only analyzed in those patients in whom all time points were available.

The association between plasma vitamin C concentration and sORP/AOC was analyzed using regression models. Linear, quadratic, logarithmic and exponential regression models were compared for the best fit by visual inspection and model *R*^2^. The association between plasma vitamin C concentration and sORP/AOC was analyzed in all available samples.

To determine whether changes in plasma vitamin C concentration, due to disease or vitamin C infusion, are reflected by changes in sORP and AOC, we used four quadrant plots to calculate the concordance between these changes. The concordance reflects the proportion of data points in which both measurements change in the same direction. To filter random error effects small changes in plasma vitamin C concentration (<5 µmol/L) were excluded.

## 3. Results

**Figure 2:**
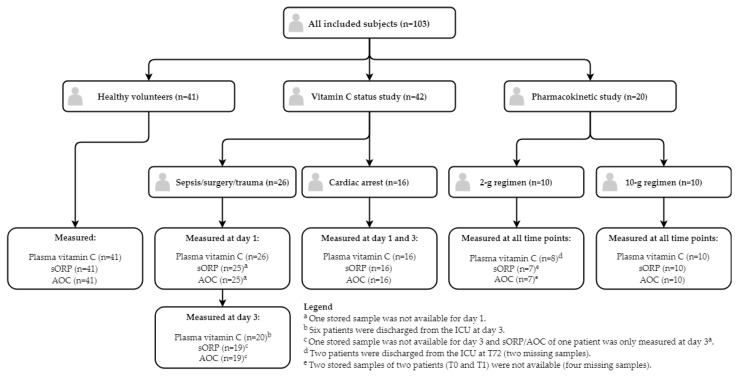
Flowchart of included subjects and sample measurements.

All included subjects and available samples in which plasma vitamin C concentration, sORP and AOC were measured are illustrated in a flowchart (Figure 2). Missing samples were due to discharge from the ICU or unavailability of previously stored samples.

Complete measurements of vitamin C, sORP and AOC were available in a total of 211 samples obtained from 41 healthy volunteers and 42 patients in the vitamin C status study and 20 patients in the pharmacokinetic study. Baseline characteristics are shown in Table 2. The healthy volunteers were younger, leaner, and more often female compared to the patients.

### 3.1. Study Measurements

#### 3.1.1. Vitamin C Status Study

**Figure 3:**
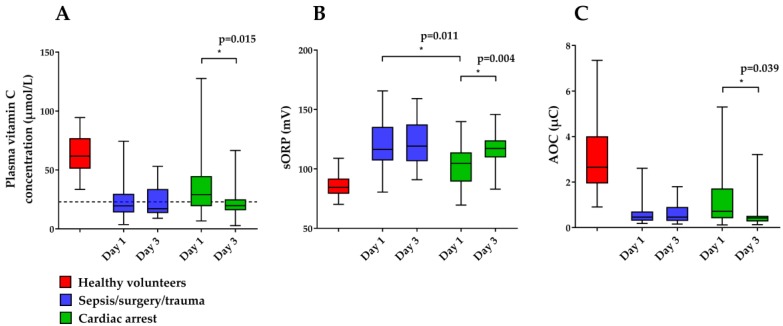
Boxplots of plasma vitamin C concentrations (**A**), static oxidation reduction potential (sORP) (**B**) and antioxidant capacity (AOC) (**C**) in the vitamin C status study at day 1 and 3. The dashed line (**A**) represents the lower limit of normal plasma vitamin C concentrations (23 µmol/L). sORP and AOC were measured in non-acidified samples.

Plasma vitamin C concentrations were available in 36/42 (85.7%) of the patients at both day 1 and 3. sORP and AOC were measured in 34 patients at both days (paired samples). Results are presented in Figure 3 and Table S1 for the different groups. Hypovitaminosis C and vitamin C deficiency (<23 µmol/L and <11 µmol/L, respectively) occurred in 0% of the 41 healthy volunteers, in 41.7% and 11.1% of the 36 patients on day 1, and in 69.4% and 16.7% of the 36 patients on day 3, respectively. In healthy volunteers, plasma vitamin C concentrations and AOC were significantly higher and sORP was significantly lower than in the patients at day 1 (*p* < 0.001 for all).

At day 1, plasma vitamin C concentrations and AOC were not different between patient groups, whereas sORP was significantly higher in the sepsis/surgery/trauma group. Between day 1 and 3, plasma vitamin C concentrations, sORP, and AOC did not change significantly in the sepsis/surgery/trauma group, while vitamin C and AOC further declined and sORP further increased significantly in the cardiac arrest patients. At day 3, no significant differences were observed between the two patient groups.

#### 3.1.2. Pharmacokinetic study

**Figure 4:**
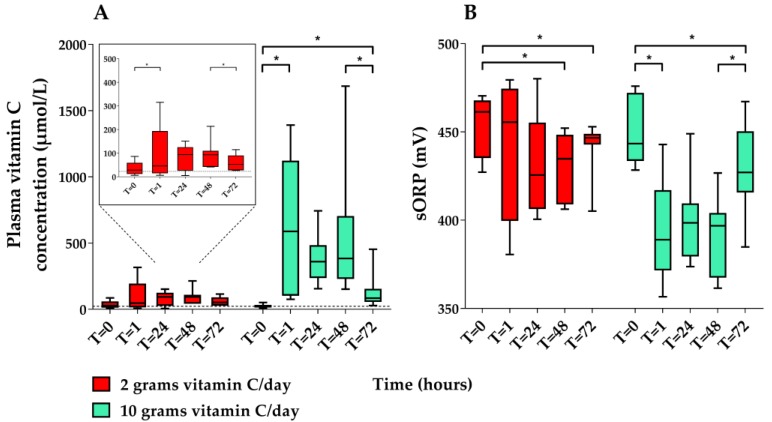
Boxplots of plasma vitamin C concentrations (**A**) and static oxidation reduction potential (sORP) (**B**) in the pharmacokinetic study at five different time points (hours). The dashed line (**A**) represents the lower limit of normal plasma vitamin C concentrations (23 µmol/L). Asterisks represent significant differences at *p* < 0.05. Vitamin C concentrations at T24 and T48 are trough concentrations. T72 represents a wash-out phase sample. sORP was measured in acidified samples.

Plasma vitamin C concentrations and sORP were available in 17/20 (85%) patients at all five time points. Seven of these patients received 2 g vitamin C per day and ten patients received 10 g vitamin C per day. Three patients in the 2-g regimen group had one or more missing samples. In total six time points were missing. The course of plasma vitamin C concentrations and sORP are presented in Figure 4 and Table S2. AOC measurements are not shown due to signal saturation at very high plasma vitamin C concentrations (see methods). In the 10-g regimen group, a large increase in plasma vitamin C concentration and a decline in sORP was seen after the start of the vitamin C infusion (T1). In the patients receiving 2 g vitamin C, plasma vitamin C concentrations increased, while sORP decreased, but these changes were less pronounced than in patients receiving 10 g vitamin C.

### 3.2. Association between Plasma Vitamin C Concentration and sORP/AOC

#### 3.2.1. Vitamin C Status Study

**Figure 5:**
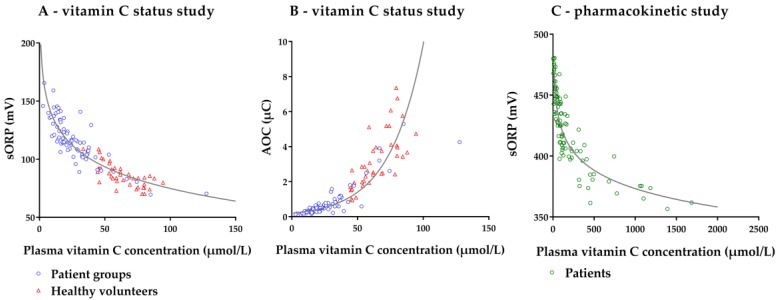
Scatter plot of the association between plasma vitamin C concentration and concomitant static oxidation reduction potential (sORP) and antioxidant capacity (AOC) in the vitamin C status study (**A**+**B**) and pharmacokinetic study (**C**). **A**: sORP = −26.89 ln(plasma vitamin C concentration) + 198.69; **B**: AOC = 0.199 × e^0.039(plasma vitamin C concentration)^; **C**: sORP = −21.74 ln(plasma vitamin C concentration) + 523.46.

To determine the association between the plasma vitamin C concentration and the concomitant sORP/AOC, we used those samples in which the three study measurements (vitamin C, sORP and AOC) were performed (*n* = 117): 41 healthy volunteers, 41 patients at day 1 and 35 patients at day 3. The association between vitamin C concentration and sORP and AOC, respectively, is shown in Figure 5. sORP was inversely associated with plasma vitamin C concentration: A negative logarithmic regression function best fitted this relation, *R*^2^ = 0.816. AOC was positively associated with plasma vitamin C concentration: An exponential regression function best fitted the relation, *R*^2^ = 0.842.

#### 3.2.2. Pharmacokinetic Study

To determine the association between the plasma vitamin C concentration and the concomitant sORP, we used those samples in which both measurements were available (*n* = 94): 44 samples obtained from ten patients in the 2-g regimen group, and 50 samples obtained from ten patients in the 10-g regimen group. The relation between vitamin C concentration and sORP is shown in Figure 5. AOC measurements are not shown due to signal saturation at very high plasma vitamin C concentrations (see methods). The negative relation between sORP and plasma vitamin C concentration was best fitted with a logarithmic regression function, *R*^2^ = 0.807.

### 3.3. Concordance between Changes in Plasma Vitamin C Concentration and Changes in ORP/AOC

#### 3.3.1. Vitamin C Status Study

**Figure 6:**
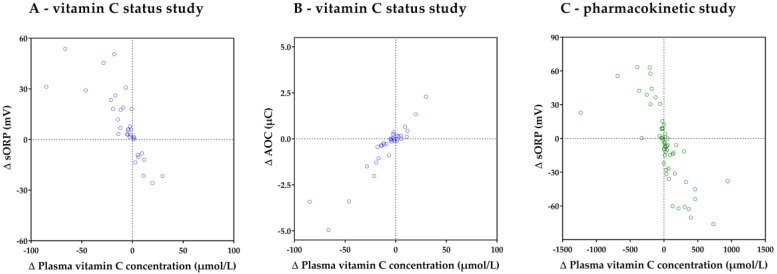
Four-quadrant plots demonstrating the concordance between the changes in plasma vitamin C concentration and changes in sORP and AOC between day 1 and day 3 in the vitamin C status study (**A** and **B** respectively), and changes in sORP per day in the pharmacokinetic study (**C**).

A four-quadrant plot, relating changes in plasma vitamin C concentration to changes in sORP and AOC between day 1 and 3, is shown in Figure 6. Changes in sORP and AOC reflected changes in plasma vitamin C concentration with a concordance of 91% and 82%, respectively. When excluding small changes in vitamin C concentration (<5 µmol/L), concordance increased to 100% and 100%, respectively.

#### 3.3.2. Pharmacokinetic Study

A four-quadrant plot, relating changes in plasma vitamin C concentration to changes in sORP, is shown in Figure 6. Differences were calculated between T0 and T24, T24 and T48, and T48 and T72. Changes in sORP reflected the changes in plasma vitamin C concentration with a concordance of 91%. When excluding small changes in vitamin C concentration (<5 µmol/L), concordance increased to 94%.

## 4. Discussion

The present study in critically ill patients and healthy volunteers demonstrates a strong negative association between the plasma vitamin C concentration and the static oxidation reduction potential (sORP) and a strong positive association with antioxidant capacity (AOC), as measured with a novel point-of-care device in plasma. sORP and AOC values followed the changes in vitamin C concentration due to disease or vitamin C infusion.

Our study is the first study determining a relation between plasma vitamin C concentration, sORP and AOC in a clinical setting. Two previous in vitro studies showed that ascorbate concentration was negatively, logarithmically related to sORP in control solutions of phosphate-buffered saline (PBS) [14,17]. This is in line with our results demonstrating the ability of the RedoxSYS system to estimate vitamin C status in patients as well. The relation between vitamin C status and AOC has not been reported before.

Our study also demonstrated a strong concordance between changes in plasma vitamin C concentration and changes in severity of oxidative stress. Vitamin C concentrations had a different course in sepsis/surgery/trauma patients compared to patients after cardiac arrest: A decrease in plasma vitamin C concentration between day 1 and 3 in the cardiac arrest patients and an already low vitamin C concentration in the patients admitted with systemic inflammation. sORP and AOC measurements followed this course. A strong concordance was also seen in the pharmacokinetic study. Patients receiving 10 g vitamin C per day immediately achieved high plasma vitamin C concentrations and sORP decreased concomitantly. The high concordance between plasma vitamin C concentration and sORP reflects the effect of high dose vitamin C in diminishing the amount of oxidative stress. This reductive effect of vitamin C has previously been shown in human plasma samples following exogenously processing [15,27]. Our study therefore suggests that sORP and AOC may be used to monitor oxidative stress and vitamin C status during the course of critical illness and during vitamin C treatment. Notable, the AOC as measured by the present device is not reliable for very high vitamin C concentrations (>500 µmol/L), but correspondence with concentrations in the (high) normal and low range was good. These are the most important ones to detect in daily clinical practice. In summary, above findings show that intravenous vitamin C treatment can reduce oxidative stress in patients’ plasma and increase plasma antioxidant capacity, which supports previous in vitro findings about vitamin C being an important circulating antioxidant [4,5].

### 4.1. Limitations

First, we measured sORP and AOC in acidified and non-acidified heparinized plasma collections that had been stored at −80 °C for several years until analysis. Even though our samples were thawed only once, a recent study showed that sORP initially decreases with 6 mV in control and 22 mV in exogenously oxidized human plasma samples after any freeze-thaw cycle before remaining constant at −80 °C [15]. However, literature showing no freeze-thaw effect is present as well [16,27]. Procedural differences might explain these differences. Second, the present study only determined the relation between vitamin C and sORP/AOC. Other antioxidants besides vitamin C may be associated with sORP and AOC as well and could have been degraded during the storage period. For example, the antioxidant glutathione is needed for the recycling of DHA and the ascorbate radical to vitamin C [28], and vitamin C recycles vitamin E. Thus, low glutathione will contribute to vitamin C deficiency and vitamin C deficiency in turn to vitamin E deficiency. Besides recycling vitamin E, vitamin C can restore oxidizing free radicals, generated from other antioxidants, with greater reduction potentials than vitamin C itself, including the hydroxyl radical, glutathione and urate [29,30]. Therefore, vitamin C can be considered as an important circulating antioxidant [5]. Moreover, vitamin C decreases serum urate concentration by decreasing urate synthesis and by increasing renal urate excretion [31]. Altogether, it is expected that vitamin C has a significant impact on the net balance of the activity between oxidants and reductants. Third, sORP measurements in acidified plasma samples (pharmacokinetic study) were higher than in non-acidified plasma. It is known that acidification influences sORP. Our preliminary prospective data show that in directly measured heparinized plasma sORP increases with ±325 mV (12 samples) after acidifying with 5.6% meta-phosphoric acid. We therefore cannot compare the absolute sORP values of the two studies. Fourth, the RedoxSYS analyzer was not able to provide reliable AOC results when plasma samples with high vitamin C concentrations (>500 µmol/L) were measured. Therefore, it seems that AOC is not suitable for monitoring vitamin C treatment in this high range. Fifth, we did not directly measure vitamin C concentrations in tissues due to ethical dilemmas and difficulties in obtaining those samples. However, intracellular vitamin C concentrations, which differ among cells [8], seem to follow plasma concentrations [8,32,33]. The validity of using plasma vitamin C concentration as an approximate of vitamin C status is supported by the finding that low plasma vitamin C concentrations are associated with impaired immunity, higher susceptibility to infections, organ failure, and mortality [2,7].

### 4.2. Strengths

This is the first clinical study showing the potential of the RedoxSYS system to estimate the plasma vitamin C concentration of critically ill patients. For this study, a large number of samples were analyzed. Importantly, the RedoxSYS analyzer is able to measure oxidative stress and antioxidant capacity in a sample volume of 30 µL in just 4 min. Therefore, the total time from sampling to test results is less than 20 min. The price of one test sensor is presently $15. Altogether, the RedoxSYS analyzer seems to be a good surrogate for the laborious and costly HPLC method, which is currently used for plasma vitamin C determination. While we cannot exclude a greater loss of vitamin C in the samples of the vitamin C status study due to longer storage, AOC measurements in our healthy volunteers’ samples were still high. This suggests that vitamin C concentrations were maintained. The literature is not consistent regarding the recommended maximum storage period for the determination of vitamin C [34,35,36]. In addition, we found no studies reporting the reliability of the sORP and AOC measurements beyond six months storage at −80 °C [16].

Up to now, the exact impact of the different collecting, processing and storage conditions on the absolute sORP and AOC values remains unknown and should be subject of future research. To define cut-off values for oxidative stress, we recommend performing fresh measurements to exclude these influences. Nevertheless, we found strong associations between vitamin C and sORP/AOC in both of our included studies, regardless of acidification of the plasma samples and storage for many years. We are currently performing a prospective study including fresh non-acidified plasma samples from critically ill patients, in which we aim to determine cut-off values for hypovitaminosis C and vitamin C deficiency. This would make sORP and AOC, as measured with the RedoxSYS system, suitable surrogate markers for the degree of oxidative stress and vitamin C status in patients.

## 5. Conclusions

This present study demonstrates a strong inverse association between the plasma vitamin C concentration and the static oxidation reduction potential (sORP) and a strong positive association with antioxidant capacity (AOC), as measured with a novel point-of-care device in plasma. sORP and AOC strongly followed changes in vitamin C status due to disease or vitamin C infusion. Concordance was high. Therefore, measuring sORP and AOC at the bedside has the potential to identify patients with vitamin C deficiency, who may especially benefit from vitamin C therapy, and to monitor oxidative stress and vitamin C status during intravenous vitamin C therapy. In order to realize these perspectives, the impact of different ways of processing blood samples has to be further elucidated.

## Figures and Tables

**Table 1:** Different sample processing conditions for each study.

	Vitamin C Status Study	Pharmacokinetic Study
Blood tubes	Heparinized	Heparinized
Centrifuging	10 min 1800 RPM	10 min 1800 RPM
Plasma processing	Not further processed before storage at −80 °C	Acidified before storage at −80 °C
Time to sORP/AOC measurements	5–6 years after storage at −80 °C	2-3 years after storage at −80 °C
Time points sORP/AOC measurements	Once for healthy subjects Day 1 and day 3 for patients	T = 0, 1, 24, 48 and 72

EDTA: Ethylenediaminetetraacetic acid; sORP: Static oxidation-reduction potential; AOC: Antioxidant capacity; T: Time (hours).

**Table 2:** Baseline characteristics.

**Vitamin C Status Study**			
**Healthy volunteers**	**Total (*n* = 41)**		
Age (years)	42 (27–49)		
Sex, male (%)	12 (29.3)		
BMI (kg/m^2^)	22.0 (20.3–25.6)		
Plasma vitamin C concentration (µmol/L) ^a^	63.3 ± 14.8		
**Patients**	**Total (*n* = 42)**	**Sepsis/surgery/trauma (*n* = 26)**	**Cardiac Arrest (*n* = 16)**
Age (years)	61 (48–76)	57 (33–76)	63 (53–77)
Sex, male (%)	27 (64.3)	15 (57.7)	12 (75.0)
BMI (kg/m^2^)	25.3 (22.4–27.6)	24.9 (22.0–26.7)	25.8 (23.3–28.2)
SOFA day 1 ^b^	7 ± 3	7 ± 4	6 ± 2
Lactate day 1 (mmol/L) ^c^	2.4 (1.7–4.9)	2.5 (1.4–5.5)	2.3 (1.7–4.9)
Plasma vitamin C concentration (µmol/L) ^a^	25.3 (16.0–36.0)	22.4 (13.6–32.9)	29.2 (19.3–44.9)
**Pharmacokinetic Study**			
**Patients**	**Total (*n* = 20)**	**2 g vitamin C/day (*n* = 10)**	**10 g vitamin C/day (*n* = 10)**
Age (years)	64 (58–78)	63 (53–78)	66 (55–77)
Sex, male (%)	13 (65.0)	6 (60.0)	7 (70.0)
BMI (kg/m^2^)	27.4 ± 5.8	29.0 ± 6.7	25.8 ± 4.7
SOFA at admission ^b^	7 (7–10)	8 (7–9)	7 (7–11)
Lactate T = 0 (mmol/L)	1.5 (1.1–1.8)	1.5 (1.0–2.2)	1.5 (1.2–1.7)
Plasma vitamin C concentration (µmol/L) ^a^	22.7 (14.7–39.5)	26.7 (14.1–47.5)	19.3 (14.3–33.0)

BMI: Body mass index; APACHE: Acute physiology and chronic health evaluation; SOFA: Sequential organ failure assessment; T: Time (hours). Data are presented as mean ± standard deviation or as median with (interquartile range). ^a^ Plasma vitamin C concentration at baseline; ^b^ SOFA-scores are calculated without the central nervous system score due to its unreliability when patients receive sedatives; ^c^ Lactate day 1 was measured in 24 patients in the sepsis/surgery/trauma group (total *n* = 40).

## Supplementary Materials

The following are available online at https://www.mdpi.com/2072-6643/11/5/1031/s1, Table S1: Plasma vitamin C concentrations and plasma sORP/AOC at day 1 and 3 of ICU-admission; Table S2: Plasma vitamin C concentrations and plasma sORP/AOC during ICU-stay.
