# Supplementary material for: Estimating Vitamin C Status in Critically Ill Patients with a Novel Point-of-Care Oxidation-Reduction Potential Measurement
Source: Nutrients. 2019 May 8;11(5):1031. doi: 10.3390/nu11051031 (PMC6566553; doi:10.3390/nu11051031)
Supplement: Supplementary file 1 [file nutrients-11-01031-s001.pdf]

nutrients

Supplementary data

Table S1. Plasma vitamin C concentrations and plasma sORP/AOC at day 1 and 3 of ICU-admission.

	**Vitamin C day 1 (µmol/l)**	**Vitamin C day 3 (µmol/l)**	**P-value**
All patients (n=34)	25.3 [16.2-36.0]	18.8 [14.5-30.2]	**0.022**
Septic/surgery/trauma (n=18)	19.6 [14.0-29.8]	17.2 [13.5-33.9]	0.472
Cardiac arrest (n=16)	29.2 [19.3-44.9]	19.8 [15.9-25.1]	**0.015**
	**sORP day 1 (mV)**	**sORP day 3 (mV)**	**P-value**
All patients (n=34)	111.4 ± 21.5	120.3 ± 16.8	**0.013**
Septic/surgery/trauma (n=18)	120.0 ± 20.8	122.1 ± 18.4	0.614
Cardiac arrest (n=16)	101.7 ± 18.3	118.2 ± 15.1	**0.004**
	**AOC day 1 (µC)**	**AOC day 3 (µC)**	**P-value**
All patients (n=34)	0.57 [0.35-1.04]	0.46 [0.27-0.68]	0.087
Septic/surgery/trauma (n=18)	0.46 [0.30-0.71]	0.46 [0.29-0.91]	1.000
Cardiac arrest (n=16)	0.72 [0.41-1.72]	0.42 [0.27-0.52]	**0.039**

sORP: Static oxidation-reduction potential; AOC: Antioxidant capacity.

Data are presented as mean ± standard deviation or as median with [interquartile range].

Table S2. Plasma vitamin C concentrations and plasma sORP/AOC during ICU-stay.

**Plasma vitamin C level**	**T=0**	**T=1**	**T=24**	**T=48**	**T=72**
Vitamin C 2 gr/day (n=7)	28.4 [12.0-59.3]	45.7 [14.6-194.1]	94.0 [26.1-125.4]	93.3 [42.3-110.0]	52.6 [27.4-90.4]
Vitamin C 10 gr/day (n=10)	19.3 [14.3-33.0]	587.6 [102.0-1121.8]	358.8 [235.9-484.6]	383.8 [229.0-703.8]	84.1 [56.0-155.4]
**sORP**	**T=0**	**T=1**	**T=24**	**T=48**	**T=72**
Vitamin C 2 gr/day (n=7)	461.3 [435.2-467.8]	455.5 [399.6-474.5]	425.5 [406.3-455.3]	434.8 [408.9-448.4]	446.6 [442.8-448.9]
Vitamin C 10 gr/day (n=10)	443.4 [433.5-472.1]	389.0 [371.6-417.0]	398.5 [379.4-409.5]	396.8 [367.4-404.1]	427.1 [415.7-450.4]

sORP: Static oxidation-reduction potential.

Data are presented as mean ± standard deviation or as median with [interquartile range].
